# Sexual and Gender Minority Representation and Experiences in Orthopaedic Surgery: A Narrative Review

**DOI:** 10.7759/cureus.110615

**Published:** 2026-06-10

**Authors:** Benjamin H Ramsahoye

**Affiliations:** 1 Trauma and Orthopaedics, Norfolk and Norwich University Hospital, Norwich, GBR

**Keywords:** gender minority, lgbt, lgbtq, orthopaedic surgery, resident, sexual minority, workforce diversity

## Abstract

Trauma and orthopaedic surgery has historically been regarded as one of the least diverse specialities in medicine. Despite increasing sexual and gender minority (SGM) populations globally, there is an apparent continued underrepresentation in orthopaedic surgery, although the existing literature is fragmented and lacks synthesis. This narrative review aims to evaluate current SGM representation in orthopaedic surgery, examine barriers to entry (including workplace experiences, prospective trainee perceptions and reports of differential treatment) and assess the impact of underrepresentation on the workforce and patient care. A literature search was conducted across PubMed, Scopus, and Google Scholar, with 28 studies selected for inclusion. Available evidence demonstrates persistently low representation of SGM individuals in orthopaedics, alongside reports of discrimination, limited visibility, and negative perceptions of the speciality among medical students and prospective trainees. These factors likely act as ongoing barriers to recruitment and retention. Emerging evidence also suggests that workforce diversity may influence patient experience and doctor-patient communication, particularly in areas involving sensitive health discussions. Addressing the representation and experiences of SGM individuals in orthopaedic surgery is important for promoting workforce equity and optimising patient care. Targeted strategies, including enhanced education, mentorship, and increased visibility of SGM clinicians, may help foster a more inclusive and diverse workforce. Further research is required to better quantify representation and evaluate the effectiveness of interventions aimed at improving diversity.

## Introduction and background

Trauma and orthopaedic surgery has historically been regarded as one of the least diverse specialities in medicine [1,2]. Several studies have investigated the representation of sexual and gender minority (SGM) groups, including lesbian, gay, bisexual, transgender and queer (LGBTQ+) individuals within orthopaedics, as well as perceptions of medical students and prospective trainees [3]. However, the current evidence base is fragmented and lacks a comprehensive synthesis of the literature.

Whilst vast improvements in diversity have been made in recent years [4], SGM groups remain underrepresented, with a likely significant impact on individuals, the workforce and patient care. This paper aims to review the current understanding of SGM representation within orthopaedics; student, trainee, and patient perceptions; and barriers to entry. The potential impact of underrepresentation on the workforce and patient care will also be reviewed. In doing so, strategies to overcome these barriers can be proposed, with the aim of improving diversity and representation in orthopaedic surgery.

This article was previously presented as a meeting abstract at the 5th International Conference on LGBT Studies on April 24, 2026.

## Review

Methods

Study Design

A narrative review of the literature was conducted to evaluate the representation of sexual and gender minority (SGM) individuals in orthopaedic surgery, with a particular focus on workforce demographics, workplace experiences, barriers to entry for prospective trainees, and the potential implications of underrepresentation for the workforce and patient care. Given the heterogeneity of study designs, populations, and reported outcomes, a narrative review methodology was selected in preference to a formal systematic review, meta-analysis, or quantitative evidence synthesis.

Search Strategy

A comprehensive literature search was conducted in PubMed, Scopus, and Google Scholar. The final search was performed on 4 May 2026. Searches were restricted to English-language publications published between January 2005 and May 2026.

The primary search strategy combined terms relating to sexual and gender minority populations with terms relating to orthopaedic surgery:

("LGBT" OR "LGBTQ" OR "sexual minority" OR "gender minority" OR "gay" OR "lesbian" OR "bisexual" OR "transgender") AND ("orthopaedic" OR "orthopedic" OR "trauma" OR "surgery" OR "surgeon" OR "resident" OR "residency" OR "workforce")

Additional searches were performed using combinations of these terms to identify literature relating to healthcare workforce diversity, workplace experiences, discrimination, mentorship, patient perspectives, and clinically relevant issues affecting SGM populations. Reference lists of included studies were manually screened to identify additional relevant publications.

Peer-reviewed studies formed the primary evidence base for this review. Selected grey literature sources, including reports from Gallup and the UK Office for National Statistics (ONS), were also reviewed where they provided population-level demographic data which allowed orthopaedic workforce representation to be contextualised. 

Eligibility Criteria

Studies were considered eligible for inclusion if they reported on the representation of SGM individuals in orthopaedics or the wider healthcare workforce, explored workplace experiences, including discrimination and inclusion, examined perceptions of medical students, trainees, healthcare professionals, or patients, or addressed SGM-related health topics relevant to orthopaedic practice. Given the relative paucity of orthopaedic-specific literature, studies examining SGM experiences within healthcare more broadly were included where relevant to the aims of the review.

Studies were excluded if they were not published in English, focused exclusively on other aspects of diversity without reference to SGM populations, or were not relevant to healthcare or orthopaedic practice.

Study Selection

Titles and abstracts were initially screened for relevance, followed by full-text review of selected articles. A total of 28 studies were included. Study selection was undertaken to provide a representative overview of the available literature addressing SGM representation, workplace experiences, barriers to entry, patient perspectives, and clinically relevant considerations within orthopaedics and healthcare more broadly.

Data Extraction and Narrative Synthesis

Data were extracted on study characteristics, including study design, population, setting, and key findings relating to SGM representation, workplace experiences, discrimination, barriers to entry, patient perspectives, and clinically relevant considerations.

Due to substantial heterogeneity in study designs, populations, and outcome measures, no pooled quantitative analysis, meta-analysis, meta-regression, or formal statistical synthesis was performed. Findings were instead synthesised using a qualitative narrative approach.

Studies were grouped into four broad thematic domains: Representation of SGM individuals within orthopaedic surgery; workplace experiences and barriers to entry; potential implications for workforce wellbeing and patient care; and strategies to improve inclusion, diversity, and representation. Findings were compared across studies to identify areas of consistency, disagreement, and gaps within the literature.

Greater interpretive weight was given to peer-reviewed studies directly examining orthopaedic surgeons, trainees, applicants, and patients. Findings derived from broader healthcare settings were interpreted cautiously and used primarily where orthopaedic-specific evidence was limited.

Assessment of Evidence

As this review sought to provide a broad overview of an emerging area of research, no formal risk-of-bias assessment tool or evidence-grading framework was applied. Instead, the strength and applicability of evidence were considered qualitatively according to study design, sample size, relevance to orthopaedic practice, and consistency of findings across the literature. Particular emphasis was placed on peer-reviewed studies directly examining orthopaedic populations.

Study Limitations

As a narrative review, this study is subject to selection bias and does not provide a quantitative synthesis of findings. No meta-analysis or formal quality assessment was performed. Consequently, conclusions are based on qualitative interpretation of a heterogeneous body of literature that includes survey-based studies, observational research, and contextual population-level reports. The limited availability of orthopaedic-specific evidence necessitated inclusion of studies from broader healthcare settings, which may reduce the direct applicability of some findings to orthopaedic practice.

Current representation of sexual and gender minority individuals

To provide demographic context, recent population-level estimates from Gallup and the UK Office for National Statistics (ONS) suggest that approximately 9% of adults in the United States identify as LGBTQ+, and that the proportion of the population identifying as SGM in the United Kingdom has also increased in recent years [5,6]. However, representation in orthopaedic surgery remains poor. The American Academy of Orthopaedic Surgeons (AAOS) identified that within their institution, only five per cent of women and three per cent of men identified as being a member of an SGM group [2]. Furthermore, the Scottish Trauma and Orthopaedics Equality Project identified that only five per cent of respondents identified as SGM [7].

There are several plausible explanations for this disparity. Many medical students, particularly those from minority groups, have reported in surveys that they perceive that orthopaedics lacks diversity [3], which in itself could present a barrier to entry. This perception may be partially attributable to stereotypes being displayed on social media. A survey of students from 1 medical school found that 86% of respondents had viewed posts on social media platforms which portrayed stereotypes of orthopaedic surgeons. These included orthopaedic surgery being “male-dominated” and for “gym bros”. For most students, these stereotypes negatively affected their view of the speciality. However, of the students who actually went on to undertake a rotation in orthopaedics, 67% felt that their experience improved their perception [8]. In another survey of medical students, perceptions of a predominantly male culture were reported to be a barrier to entry [9], and this may put off SGM doctors from pursuing a career in the speciality. Moreover, from their single-centre survey at a state medical school, Rosecrance et al. found orthopaedic surgery to be reported as the least welcoming speciality to SGM individuals of all, even more so than other surgical specialities, including neurosurgery and general surgery [10].

Experiences of sexual and gender minority individuals in the orthopaedic workforce

Several reports of maltreatment of SGM individuals in orthopaedics may explain why these perceptions exist. A survey conducted by Pride Ortho, a community of SGM orthopaedic surgeons and allies, found that almost one-third of SGM respondents were only partially out and open with their identity at work. Moreover, 74% of respondents from SGM groups reported having experienced some form of bullying or discrimination, compared with zero per cent of heterosexual respondents [11]. Another study surveying applicants to orthopaedic training identified that 61% of SGM applicants had been subject to hurtful language at some point during orthopaedic rotations and research activities. Overall, they were approximately four times more likely to experience hostility and five times more likely to experience discrimination [12]. Douglas Weaver spoke of his experience as an openly gay subintern, where theatre staff made offensive comments about the appearance of a transgender patient whilst they were under anaesthesia, and how he felt that he could not speak up in that environment. He also discussed how he had to make careful considerations when applying to training programmes as an SGM individual, regarding which programmes or locations would be most accepting [13].

Impact of underrepresentation on the workforce and patient care

Maltreatment, bullying, and discriminatory behaviours directed towards SGM individuals, as well as the other barriers to entry discussed, partially explain the ongoing underrepresentation of SGM within orthopaedics. This impact on workforce diversity may contribute to reduced workforce wellbeing and productivity. Although orthopaedic-specific evidence is limited, recent reports state that SGM doctors are more likely to face mental health issues and burnout [14].

Several studies have also highlighted the importance of workforce diversity in optimising patient care. Abghari et al. surveyed patient preferences for various attributes of theoretical orthopaedic surgeons. They found that generally, patients did not have a preference when it came to age, race, sex and gender. However, for the minority of patients who did, they tended to prefer surgeons of a similar demographic to themselves [15]. Additionally, in a broader healthcare study that explored health inequalities faced by sexual minority men (SMM), semi-structured qualitative interviews found that patient-centred care was important in allowing patients to have agency and participate in decision-making around their own health, with many participants in agreement that this was more likely to be delivered by SGM identifying providers [16]. Furthermore, a study by Gioia et al. identified that SMM were more likely to discuss topics relating to sexual health when they perceived their healthcare provider to be gay or bisexual [17].

Whilst some of these studies did not specifically survey orthopaedic patient populations, they may have implications for orthopaedic practice, particularly where sensitive sexual health histories are relevant. The Centers for Disease Control and Prevention (CDC) have identified that the transmission of gonorrhoea is significantly higher in men who have sex with men (MSM) compared with heterosexuals [18]. Disseminated gonococcal infections have an increasing prevalence, with many presenting with septic arthritis [19,20]. As broader healthcare studies have highlighted, improved representation of gay and bisexual men in the workforce can improve doctor-patient relations and promote open discussions regarding sexual health topics [17]. In an orthopaedic population, improved doctor-patient communication may result in early intervention and prevention of certain complications of gonococcal septic arthritis, including joint destruction [21]. However, evidence of these improved communication practices on orthopaedic outcomes is indirect and warrants further investigation.

Another important area in orthopaedics regarding SGM healthcare is the effect of hormone blockers on bone health. Their use in pre-pubescent individuals remains an area of controversy in many countries, with several studies showing that the use of GnRH analogues during adolescence results in reduced bone mineral density at a critical period for skeletal development [22,23]. Although further research is required to investigate the implications of this in terms of fracture risk, it remains an important area for orthopaedic surgeons to be aware of. With many transgender patients expressing the importance of having gender and sexual-orientation-tolerant healthcare providers [24,25], having a workforce which represents this population is important.

Strategies to improve inclusion, diversity and representation of sexual and gender minority groups

Given the current evidence of underrepresentation in orthopaedic surgery and the potential impact on individuals, the workforce and patient care, targeted strategies are required to meaningfully improve diversity and representation. One method may be to place increased emphasis on SGM-related topics during undergraduate medical education. Obedin-Maliver et al. conducted a study of 176 medical schools, finding that the median time dedicated to these topics was only five hours across the entire course [26]. Dedicating additional time to these topics may create a more inclusive and welcoming environment, attracting diversity into the speciality. Additionally, increasing exposure to orthopaedic surgery during undergraduate clinical placements may alter student perceptions and stereotypical views, as demonstrated by Bhatti et al. [8].

Mentorship programmes offer an alternative strategy to increasing SGM representation [27]. A survey of SGM health professional trainees found that 72% of respondents valued having an SGM-identifying mentor, with 59% stating that they felt this was important for their career development [28]. Whilst this study was not specific to orthopaedic surgery, the introduction of mentorship programmes within orthopaedic departments and societies may entice prospective SGM trainees towards the speciality. 

Lastly, it is important that SGM individuals are visible within the workforce. International communities and initiatives such as “Pride Ortho” play an important role in increasing the visibility of SGM surgeons. The continuing development of such communities, in combination with local initiatives to increase visibility, will make orthopaedics a more welcoming and less intimidating speciality for prospective trainees.

## Conclusions

Sexual and gender minority individuals remain relatively underrepresented in the orthopaedic surgery workforce. Persistent barriers, including limited visibility, entrenched stereotypes, and reported workplace discrimination, continue to shape perceptions of the speciality. It is important to address these barriers, not only for workforce equity, but also because there is potential for improving patient care in an increasingly diverse population.

There remains a lack of major epidemiological data regarding SGM representation in orthopaedics. Further multi-centre surveys and cross-sectional studies are warranted to ascertain a more accurate understanding of current representation. Furthermore, the impact of potential strategies to improve representation, including educational initiatives and mentorship programmes, needs to be explored, as evidence has mostly been derived from broader healthcare settings. In doing so, it is plausible that orthopaedic surgery can continue to become a more equally represented and inclusive speciality, promoting workforce productivity and improved standards of patient care.

## References

[1] Lin JS, Lattanza LL, Weber KL, Balch Samora J (2020). Improving sexual, racial, and ethnic diversity in orthopedics: an imperative. Orthopedics.

[2] Feroe AG, Odum SM, Samora JB (2024). What is the representation of sexual and gender minority identities among orthopaedic professionals in the United States?. Clin Orthop Relat Res.

[3] Rahman R, Zhang B, Humbyrd CJ, LaPorte D (2021). How do medical students perceive diversity in orthopaedic surgery, and how do their perceptions change after an orthopaedic clinical rotation?. Clin Orthop Relat Res.

[4] Daniels EW, French K, Murphy LA, Grant RE (2012). Has diversity increased in orthopaedic residency programs since 1995?. Clin Orthop Relat Res.

[5] (2026). LGBTQ+ identification in U.S. rises to 9.3%. https://news.gallup.com/poll/656708/lgbtq-identification-rises.aspx.

[6] (2026). Sexual orientation, UK: 2024. UK.

[7] Jabbal M, Cherry J, Eastwood D, Scott CE, Walmsley P, Baird E (2025). STEP 1: The Scottish Trauma &amp; Orthopaedics Equality Project. Demographics and working patterns of a national workforce. Bone Jt Open.

[8] Bhatti NS, Sheldon A, Nah IY, Shahzad H, Goyal K, Yu E (2025). Perceptions of orthopaedics and the effect of stereotypes on medical students. JB JS Open Access.

[9] Entezari B, Kerslake S, Mann S, Wolfstadt JI, Hopman W, Ferguson PC, Hiemstra LA (2025). Medical student perceptions of the barriers to entering orthopaedic surgery differ by gender. Clin Teach.

[10] Rosecrance K, Archibald A, Victor R, Lasso ET, Nore C, Barrios C (2023). Medical student perspectives on sexual and gender minority acceptance in surgical specialties and sexual and gender minority education. J Surg Res.

[11] Kumaran Y, Bellamy J, Maciejewski R, Tulchin-Francis K, Samora JB (2024). How much bullying and discrimination are reported by sexual and gender minorities in orthopaedics?. Clin Orthop Relat Res.

[12] Weaver DJ, Dagher T, Duong N, Winfrey S, Koo A, Balach T (2025). Assessing the experiences of sexual and gender minority applicants to orthopaedic surgery residency. JB JS Open Access.

[13] Weaver DJ (2022). What’s important: applying to orthopaedic surgery as a member of the LGBTQ community. J Bone Joint Surg Am.

[14] Streed CG Jr, Navarra M, Halem J, Stewart MT, Rowe SG (2024). Academic physician and trainee occupational well-being by sexual and gender minority status. JAMA Netw Open.

[15] Abghari MS, Takemoto R, Sadiq A, Karia R, Phillips D, Egol KA (2014). Patient perceptions and preferences when choosing an orthopaedic surgeon. Iowa Orthop J.

[16] Hascher K, Jaiswal J, LoSchiavo C (2024). Lack of informed and affirming healthcare for sexual minority men: a call for patient-centered care. J Gen Intern Med.

[17] Gioia SA, Russell MA, Zimet GD, Stupiansky NW, Rosenberger JG (2021). The role of disclosure &amp; perceptions about providers in health discussions among gay and bisexual young men. Patient Educ Couns.

[18] Fairley CK, Hocking JS, Zhang L, Chow EP (2017). Frequent transmission of gonorrhea in men who have sex with men. Emerg Infect Dis.

[19] Nossent J, Raymond W, Keen H, Preen DB, Inderjeeth CA (2022). Septic arthritis due to Neisseria gonorrhoea in Western Australia. Intern Med J.

[20] Tang EC, Johnson KA, Alvarado L (2023). Characterizing the rise of disseminated gonococcal infections in California, July 2020-July 2021. Clin Infect Dis.

[21] Matsuo T, Khodadadi RB, Lahr BD (2025). Consequences of delaying surgical intervention in patients with native joint septic arthritis. Open Forum Infect Dis.

[22] Schagen SE, Wouters FM, Cohen-Kettenis PT, Gooren LJ, Hannema SE (2020). Bone development in transgender adolescents treated with GnRH analogues and subsequent gender-affirming hormones. J Clin Endocrinol Metab.

[23] van der Loos MA, Vlot MC, Klink DT, Hannema SE, den Heijer M, Wiepjes CM (2023). Bone mineral density in transgender adolescents treated with puberty suppression and subsequent gender-affirming hormones. JAMA Pediatr.

[24] Poteat T, German D, Kerrigan D (2013). Managing uncertainty: a grounded theory of stigma in transgender health care encounters. Soc Sci Med.

[25] Lifshitz D, Yaish I, Wagner-Kolasko G (2022). Transgender men's preferences when choosing obstetricians and gynecologists. Isr J Health Policy Res.

[26] Obedin-Maliver J, Goldsmith ES, Stewart L (2011). Lesbian, gay, bisexual, and transgender-related content in undergraduate medical education. JAMA.

[27] Chu A, Lin JS, Moontasri NJ, Hammouri Q, Samora JB (2022). LGBTQ+ in orthopaedics: creating an open and inclusive environment. J Am Acad Orthop Surg.

[28] Sánchez NF, Callahan E, Brewster C, Poll-Hunter N, Sánchez JP (2018). The future LGBT health professional: perspectives on career and personal mentorship. LGBT Health.

